# Energy-Efficient Nonuniform Content Edge Pre-Caching to Improve Quality of Service in Fog Radio Access Networks

**DOI:** 10.3390/s19061422

**Published:** 2019-03-22

**Authors:** Yi Cen, Yigang Cen, Ke Wang, Jingcong Li

**Affiliations:** 1 School of Information Engineering, Minzu University of China, Beijing 100081, China; yi_cen@126.com (Y.C.); jcli@pku.org.cn (J.L.); 2 School of Computer and Information Technology, Beijing Jiaotong University, Beijing 100044, China; ygcen@bjtu.edu.cn; 3 School of Information and Communication Engineering, Beijing University of Posts and Telecommunication, Beijing 100876, China

**Keywords:** fog radio access network, non-uniform mobile edge caching, preference inference, group partition, non-convex matrix/tensor completion

## Abstract

The fog radio access network (F-RAN) equipped with enhanced remote radio heads (eRRHs), which can pre-store some requested files in the edge cache and support mobile edge computing (MEC). To guarantee the quality-of-service (QoS) and energy efficiency of F-RAN, a proper content caching strategy is necessary to avoid coarse content storing locally in the cache or frequent fetching from a centralized baseband signal processing unit (BBU) pool via backhauls. In this paper we investigate the relationships among eRRH/terminal activities and content requesting in F-RANs, and propose an edge content caching strategy for eRRHs by mining out mobile network behavior information. Especially, to attain the inference for appropriate content caching, we establish a pre-mapping containing content preference information and geographical influence by an efficient non-uniformed accelerated matrix completion algorithm. The energy consumption analysis is given in order to discuss the energy saving properties of the proposed edge content caching strategy. Simulation results demonstrate our theoretical analysis on the inference validity of the pre-mapping construction method in static and dynamic cases, and show the energy efficiency achieved by the proposed edge content pre-caching strategy.

## 1. Introduction

**Figure 1:**
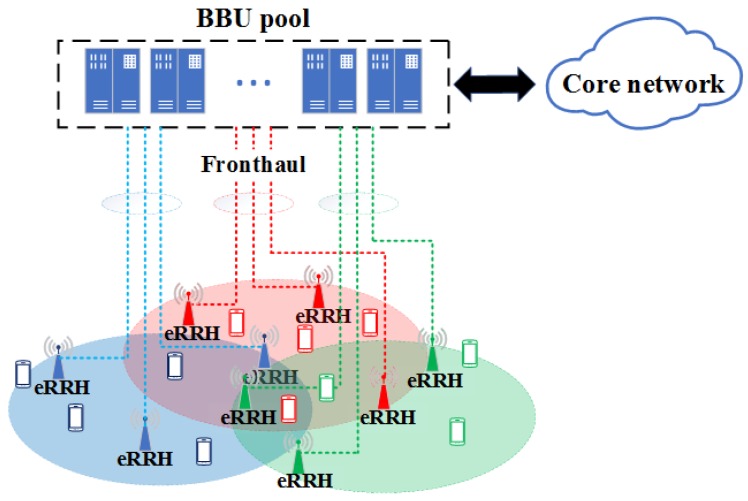
An architecture of F-RAN scheduling framework. The UEs with the same preference are denoted by the same color and the dotted loops of different colors denote the areas where the corresponding preferences are dominant.

Recently, the fog radio access network (F-RAN) has been proposed as an emerging network architecture of a cloud radio access network (C-RAN) for fifth generation wireless systems (5G), which aims to address the limitations of previous cellular standards and be a prospective key enabler for future Internet-of-Things (IoT) [1]. In a typical C-RAN, a centralized baseband signal processing unit (BBU) pool is equipped for baseband processing of a remote radio heads (RRHs) set, connected to the BBUs by fronthaul links, to save on operational expenditures and reduce energy consumption [2,3,4,5]. Although some efficient signal compression methods have been proposed for C-RANs [6], it is insufficient to satisfy the dramatically increasing requirements from mobile users for real-time services with high quality-of-service (QoS) guarantees. To address this challenge, the enhanced architecture of F-RAN allowing the RRHs with the capability of storage and signal processing functionalities, was proposed [7,8,9]. With the enhanced RRHs (eRRHs), edge caching can be performed to pre-fetch some requested files to the eRRHs local caches (as illustrated in Figure 1). Subsequently, the burden on backhaul is relieved and higher spectral efficiency or lower delivery latency can be obtained for users’ requesting cached files from incorporating caching units. In general, the goal of F-RAN architecture is to optimize the system performance in terms of delivery rate by leveraging both BBU and edge caching, which is different from that in C-RAN.

As a cache-aided system, F-RAN operates in the pre-fetching and delivery phases [10,11,12]. The pre-fetching normally stores the content by constant popularity ranking in the large time scale corresponding to multiple transmission intervals. Instead, the delivery phase operates separately on each transmission interval based on the cached file messages. Several recent studies involved the scheme and the performance of F-RAN in this context. In [7,10] the fronthaul-aware design via the pre-fetching policy was studied to minimize the average delivery latency with the cache memory constraints, and in [13] the optimal caching and delivery strategies that minimize the delivery latency are characterized for system designing. In [12,14], trade-off between the total power consumed and the total backhaul capacity needed in the downlink of the cache-aided RAN was studied. It has shown that the energy consumption is decreased due to the increase of spatial diversity by cooperative communication, meanwhile the backhaul burden is increased due to the delivery of uncached files to more eRRHs. The aforementioned research indicates that the content edge-storage strategy design plays a critical role in improving the performances of F-RAN.

Designing content edge caching strategies to more closely meet the needs can greatly alleviate the burden on backhauls, and reduce the delay and energy consumption for a large number of users. Notice that caching popular files can make the design of the delivery phase more meaningful and sensible to meet the needs of most users; almost all the content edge-storage strategies via pre-fetching were directly proposed due to this most-popular-caching premise. To the best of our knowledge, the content edge-storage strategies in the literature are considered to be based on an assumption that all files are available at the BBU and the popularity of the file is modeled by Zipf distribution [15]. More specifically, for Zipf distribution, let the files be labeled in the order of popularity from the most to the least popular ones, such that the most popular file has index f=1 and the least popular file has index f=F. Then the probability of a requested file $f\in \left\{1,2,\ldots ,F\right\}$ is $P\left(f\right)=cf^{-s}$ satisfying $\sum_{f=1}^{F}P\left(f\right)=1$. Therefore, consider that user equipments (UEs) in an F-RAN are served by multiple eRRHs that are connected to a BBU pool through digital fronthaul links, UE${}_{k}$ selects file $f_{k}\in \left\{1,2,\ldots ,F\right\}$ with the probability $P\left(f_{k}\right)$, and the requested files $f_{k}$ are independent across the index *k*. The increasing of the exponent *s* makes the probability of selecting a small group of files larger. When the parameter *s* is adjusted appropriately, only a few popular files are frequently requested by UEs.

Although the above content selecting criterion can simplify the discussion, the real situation of the content obtaining in F-RANs is much more complex due to the users’ social and activity limitation, which has noticeable impact on the performances of F-RAN. For instance, there are usually some certain activity regions for different users, which endows the content request of the individual with regional features. Also, the preferences of the users in different districts, such as the area around the school, the airport or the mall, are often relatively different, which can make the content distribution non-uniformed for different groups. Moreover, for some multimedia users, the obtained information is inevitably lagging behind once the cached contents are not desired by them. It means that judging which content to be cached from past requesting actions is reasonable and necessary in order to guarantee the QoS. It is shown that such contradictions are particularly prominent under the condition of limited edge cache in eRRH, and a straightforward way to utilize Zipf distribution for content caching is inappropriate to realistic needs. However, notice that the massive data on the activity of request is recorded and the powerful computational capacity is provided by the computing resources of the cloud, it is possible to design more efficient content-obtaining strategies relying on these foundations.

With this consideration, in this paper, we propose a caching content selection strategy by digging out users’ network behavior information and improving the distribution on content allocation. We analyze the relationship between scope of eRRH/UEs’ activities and content requests in an F-RAN, and then establish pre-mapping inferred by an efficient matrix completion algorithm for an appropriate content edge pre-caching. Especially, the proposed matrix completion algorithm gets better at the accuracy in the case of non-uniform data sampling and computational efficiency. Furthermore, we analyze the energy consumption of the content edge pre-caching strategy based on the proposed pre-mapping. Numerical results are provided to prove the effectiveness of the given inference method for the pre-mapping construction in static and dynamic cases, and illustrate the energy saving characteristics of the content edge pre-caching.

This paper is structured as follows. In Section 2 we introduce the system model considered and state the caching file selection strategy. The data structure corresponding to the UEs’ requests for the entire researched F-RAN is established mathematically as well. in Section 3, the optimization problem of the content edge caching pre-mapping construction is presented and the non-uniform non-convex matrix completion algorithm is proposed for solving the problem. The corresponding energy consumption analysis for the caching content selection strategy is provided in Section 4. Simulation results of the algorithm and the energy consumption performances are shown in Section 5, followed by the conclusion in Section 6.

## 2. System Model and Problem Statement

We consider the downlink transmission of an F-RAN as illustrated in Figure 1. *N* eRRHs are deployed in the network and cooperatively serve all users. Each eRRH, equipped with *L* antennas and a cache of the same size, can connect to the BBU pool via individual backhaul links with finite capacity. The cluster-scale joint management, such as scheduling and resource allocation, can be implemented. On the other hand, UEs are uniformly and independently distributed within the network. In each scheduling interval, *K* users will be scheduled, and send their content requests according to some preferences. Assume that each user can request at most one content at its scheduled time and the content cache in BBU pool stores the set of all content objects required by the users, which is denoted as a set $S_{c}=\left\{F_{1},\ldots ,F_{C}\right\}$, with $F_{1},\ldots ,F_{C}$ all of the same size and belonging to *I* types. Then in their own activity regions, UEs requesting the contents that belong to the same subset $S_{m}\subset S_{c}$ (with the same color icon shown in Figure 1) can form a multicast UE cluster $G_{m}$. The *m*-th cluster $G_{m}$, limited by the preferences and activity regions, is served cooperatively by a cluster of eRRHs, which is denoted as $R_{m}$. It should be noted that, although the eRRH clusters serving UE clusters can overlap with each other, the overlap of the eRRH clusters is relatively small and irregular since the service area of an eRRH is limited and the eRRH equipments are deployed in the sufficiently large area. With this consideration, we ignore this characteristic here to simplify the analysis and assume that the users in the overlap are served by only one eRRH cluster with high probability, i.e., $G_{m}\cap G_{m^{\prime }}=\emptyset$ and $R_{m}\cap R_{m^{\prime }}=\emptyset$, $m\neq m^{\prime }$.

**Figure 2:**
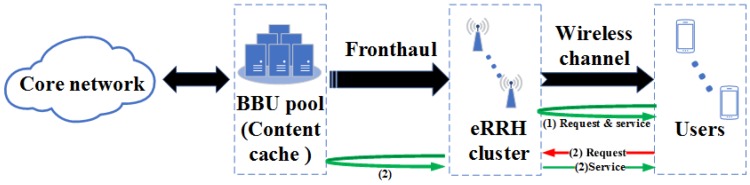
Queueing model of content object transmission with one eRRH-cluster content caching in F-RANs.

**Content caching and transmission:** Without loss of generality, we set K>N and focus on a typical eRRH/UE cluster, i.e., $G_{m}$ and $R_{m}$ (represent by the icons with the same color in Figure 1). While the content cache of BBU pool is deployed to fully exploit the potential of content caching in the F-RAN, the content cache of each eRRH in the same cluster only contains the contents that are most likely to be requested by the users in the same area. The content requests from served users are aggregated at the edge eRRH cluster in the F-RAN, and can be treated as follows: First, the eRRH cluster checks its cluster content caches and the requests can be served immediately if the desired content is available at the caches (illustrated as the procedure (1) in Figure 2). Otherwise, the requests will be forwarded to the BBU pool content cache, and then the corresponding content can be provided through the fronthaul link from the BBU pool. Then the requests can be handled similarly to the case in which the content resides in the content caches in the cluster (illustrated as the procedure (2) in Figure 2).

To increasing the operability, the preferences of the UEs’ requests and the corresponding contents will be classified into different types. For each type, the contents will be sorted according to the timeliness and the popularity, and pre-cached in the eRRHs according to the UEs’ preferences inferred by the requests. Because the caching via the preferences of the individual can reduce the probability of the procedure (2) execution, which can lead to improved QoS guarantees with low power consumption in practical F-RANs, it is reasonable to propose an eRRH content per-caching strategy depending on the UEs’ behaviors.

**Content preference data model:** Note that the restricted mobility and the randomness of request for UEs in line with the assumptions at the beginning of this section, we establish the content preference data model associated with the UEs’ requests based on the following setting: (i) the users usually stay in some limited areas depending on the occupation and habit, etc. This results in that only a limited part of the eRRHs (compared with the overall scale in the researched F-RAN) can serve the certain UEs; (ii) Due to the restricted mobility of each UE, the eRRH in the service cluster to receive the UE’s request can be deemed to be selected randomly. This means that some eRRHs in the serving cluster $S_{m}$ may not obtain the request of the UE in the $G_{m}$; (iii) It is also obvious that the number of content types interested in is far less than the scale of the users. Hence, with these prerequisites, we first let *j* and *k* denote the indices of eRRHs and UEs respectively, the basic dataset reflecting the content types that the UEs’ requests and can be structured as the preference matrix $A\in {\left\{a_{1},a_{2},\ldots ,a_{I},0\right\}}^{N\times K}$ with the entry as follows:

(1) $$A_{jk}=\left\{\begin{array}{c} \varphi_{m,i}\;\;\;\mathrm{if}\;k\;\mathrm{is}\;\mathrm{served}\;\mathrm{by}\;\mathrm{the}\;j\text{-}\mathrm{th}\;\mathrm{eRRH}\;\mathrm{belonging}\;\mathrm{to}\;R_{m}\;\mathrm{and}\;\mathrm{has}\\ \;\;\;\;\;\;\;\;\;\;\mathrm{sent}\;\mathrm{the}\;\mathrm{request}\;\mathrm{to}\;j\;\mathrm{for}\;\mathrm{the}\;\mathrm{content}\;\mathrm{in}\;\mathrm{the}\;i\text{-}\mathrm{th}\;\mathrm{type},\\ 0\;\;\;\;\;\;\;\;\mathrm{if}\;\mathrm{the}\;\mathrm{relationship}\;\mathrm{between}\;k\;\mathrm{and}\;j\;\mathrm{does}\;\mathrm{not}\;\mathrm{exist}, \end{array}\right.$$

where $\varphi_{m,i}$ is a positive integer and $\varphi_{m,i}=a_{i}\in \left\{a_{1},a_{2},\ldots ,a_{I}\right\}$. The value of $a_{i}$ can be set as an arbitrary integer for simplicity provided that there is no popular rank. However, if the popular rank exists, the value of $a_{i}$ will be set according to the way used for Zipf distribution setting and refer to the request history of the corresponding user.

**Figure 3:**
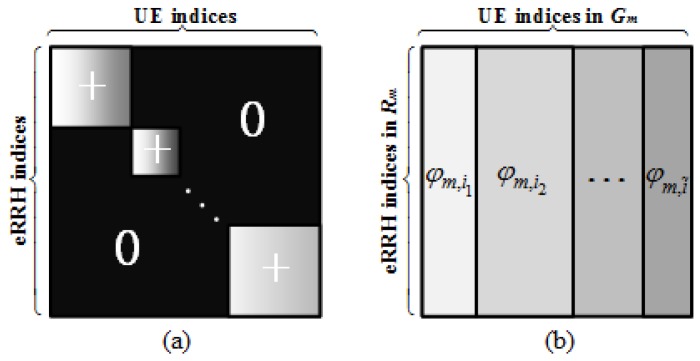
The structure of matrix **A** corresponding to the complete request data for the entire F-RAN. (**a**) presents the structure of the entire matrix; (**b**) is used to illustrate each positive sub-block with different shades of gray, the rows and the columns of the sub-block correspond to the users and the eRRHs in the same cluster.

We now show that the preference matrix **A** with the complete request dataset is low rank. For UE *k*, the entries $A_{jk}$ corresponding to the *m*-th eRRH cluster $R_{m}$ are assigned as the same $a_{i}$ since all eRRHs in the cluster should serve UE *k*. Thus, with a suitable realignment of users and eRRHs, A can be represented as a block-diagonal matrix where the entries within the diagonal blocks are positive and the others within the off-diagonal blocks are all 0’s. Furthermore, for each “positive” block, all entries of the diagonal sub-blocks are $\varphi_{m,i}$ (shown in Figure 3). The following theorem provides the upper bound for the rank of the complete preference matrix.

**Theorem** **1.** (Low rank structure of request preference data model): *The complete preference matrix A$\in {\left\{a_{1},a_{2},\ldots ,a_{I},0\right\}}^{N\times K}$ has rank $r=N_{e-ug}I$ at most, where $N_{e-ug}$ and I correspond to the number of multicast eRRH/UE clusters and that of content types respectively.*

**Proof.**  Suppose that all clusters of eRRHs are in service, then the eRRHs set can be divided into $N_{e-ug}$ clusters, i.e., $R_{m}$, $m=1,\ldots ,N_{e-ug}$. Further, for each eRRH/UE cluster, the UEs of $G_{m}$ can be classified into several common preference sub-groups, denoted by $G_{m(i)}$ and the number of the sub-group $G_{m(i)}$ in the entire network is no more than $N_{e-ug}I$. After suitable reordering indices of nodes (UEs) in $G_{m(i)}$, the corresponding row vector of A are all identical to the following form

(2) $$a=\left(0,\ldots ,0,\underset{\mathrm{the}\;m\mathrm{th}\;\mathrm{cluster}}{\underset{︸}{\varphi_{m,i_{1}},\ldots ,\varphi_{m,i_{1}},\ldots ,\varphi_{m,i_{d_{m}}},\ldots ,\varphi_{m,i_{d_{m}}}}},0,\ldots ,0\right),$$

where $d_{m}$ is the cardinality of the requested content type subset $\left\{i_{1},\ldots ,i_{d_{m}}\right\}$ in $G_{m}$ and $d_{m}\leq I$. Then let us take all UE subgroups into account, we operate column vectors of the m(i) sub-group by the column elementary transformation, which is equivalent to subtracting the first column vector of the *i*-th sub-group from the other ones, etc. Do the similar operations for row vectors of A and rearrange the rows and columns of A, we obtain

(3) $$A\to \left(\begin{array}{cc} \Phi & 0\\ 0 & 0 \end{array}\right)\;\mathrm{and}\;\Phi =\left(\begin{array}{cccc} \tilde{\varphi } & 0 & \cdots & 0\\ 0 & \tilde{\varphi } & \cdots & 0\\ \vdots & \vdots & \ddots & \vdots \\ 0 & 0 & \cdots & \tilde{\varphi } \end{array}\right),$$

where $\Phi \in R^{r\times r}$ and rank(A)=rank(Φ). $\tilde{\varphi }$ denotes arbitrary $a_{i}$ for convenience. It is easy to deduce that $rank\left(\Phi \right)\leq N_{e-ug}I$, therefore $rank\left(A\right)\leq N_{e-ug}I$. □

Despite of a clear structure retained by complete preference matrix **A**, the restricted mobility and the randomness of request for UEs (i.e., the Prerequisite (i) and (ii)) lead to a serious observation missing on the complete request preference data (the preference matrix **A**). The more likely situation is that a very limited number of requests are recorded and can be indicated by $P_{\Omega }\left(A\right),$ where Ω is the index set of the recorded entries, $P_{\Omega }$ denotes the orthogonal projection operator onto the span of matrices vanishing outside of Ω, so that the (*j*,*k*)-th component of $P_{\Omega }\left(A\right)$ is equal to the (*j*,*k*)-th component of A when $\left(j,k\right)\in \Omega$ and zero otherwise. Especially, for each row (column) of A, we find that the number of non-zero entries in the row indicates the activity of the corresponding UE. Therewith, we denote the number of non-zero entries in the *k*th column as $N_{k}$, and then define the activity of the *k*th UE as q˜k=NkNkNΩNΩ, where $N_{\Omega }=\left|\Omega \right|$ represents the number of observed non-zero entries in A. The activity vector of all UEs can be further defined as $q={\left({\tilde{q}}_{1},\ldots ,{\tilde{q}}_{K}\right)}^{T}$. Similarly, the activity of eRRH means the measurement that the eRRH caches the proper contents and serves UEs. The activity vector of all eRRHs can also be defined as $p={\left({\tilde{p}}_{1},\ldots ,{\tilde{p}}_{N}\right)}^{T}$ where p˜j=NjNjNΩNΩ ($N_{j}$ is the number of non-zero entries in the *j*th row). It is observed that activity brings out the non-uniform observations/samples.

With these known conditions, we define the content edge-caching pre-mapping construction as preference data matrix inferring. More specifically, the core mission is inferring the unknown potential relationships between the UE and eRRH in the F-RAN, and further partitioning the cluster according to the criterion associated with preference and randomness of requests in active area. On this basis, **our eRRH content per-caching strategy** is designed as follows: Guided by the entries of the inferred preference matrix (i.e., the pre-mapping), BBU pool selects the most popular content of each type according to the inferred results, and then pre-sends contents to several alternative eRRHs for the corresponding UE cluster service. Besides, if the eRRH cache exists in free space, the most popular contents except the pre-sent ones will be transmitted to the eRRH until no space left in the edge-cache. Due to the prediction, this strategy based on pre-mapping seems to satisfy the users’ needs with high probability and meaningful in the scenario such as the super-resolution videos pre-caching for high throughput transmission QoS and low energy consumption.

## 3. Nonuniform Pre-Mapping Construction via Nonconvex Optimization

In this section, on the basis of Theorem 1 and properties aforementioned, we establish a non-uniform pre-mapping to infer the unknown potential preference data in the F-RAN and further achieve eRRH/UE cluster partition according to the structure of the preference matrix. Since the complete preference matrix **A** obeys the low-rank structure, it is reasonable to utilize the low-rank matrix completion [16,17,18] to achieve pre-mapping construction. However, main methods based upon low-rank matrix completion algorithms are assumed that the data are sampled under uniform distribution. This does not hold for our scenario and system model, owing to the close relationship between the preference data distribution and the distinct activity levels of participants. To overcome the obstacle of non-uniform observation, we propose the non-convex matrix completion with iteratively re-weighted modified trace norm regularization for clustering:

Given a distribution $P_{jk}$ (j=1,…,N and k=1,…,K) that reflects the UEs’ activity of requesting, for the objective matrix $X\in {\left\{a_{1},a_{2},\ldots ,a_{I},0\right\}}^{N\times K}$, the modified trace norm of $X\in R^{N\times K}$ is defined as ${∥X∥}_{*\left(p,q\right)}={∥diag{\left(p\right)}^{1/2\;}Xdiag{\left(q\right)}^{1/2}∥}_{*}.$ Here, ${∥•∥}_{*}$ denotes the trace norm, $\sigma_{\tilde{i}}\left(diag{\left(p\right)}^{1/2}Xdiag{\left(q\right)}^{1/2}\right)$ denotes the $\tilde{i}$-th singular value of the matrix and redesignated as $\sigma_{\tilde{i},\left(p,q\right)}$ for simplicity. $diag{\left(p\right)}^{1/2}$ is the diagonal matrix with the activity vector of all participants: $p={\left(p_{1},\ldots ,p_{j},\ldots ,p_{N}\right)}^{T}$, where $p_{j}$ represents the row marginal, i.e., $p_{j}=\sum_{k=1}^{K}P_{jk}$. Similarly $diag{\left(q\right)}^{1/2}$ corresponds to the columns of X and possesses similar relations (i.e., $q_{k}=\sum_{j=1}^{N}P_{jk}$) (see Section 2). With this definition, matrix completion with modified trace norm regularization involves the following optimization

(4) $$\underset{X}{\arg\min}{∥X∥}_{*\left(p,q\right)}\;s.t.\;P_{\Omega }\left(X\right)=P_{\Omega }\left(A\right).$$

Note that compared to $l_{1}$ norm, the $l_{\vartheta }$ quasi-norm, 0<ϑ<1, makes a closer approximation to the counting norm $l_{0}$, which is the number of nonzero entries of *x*, the variants of non-convex $l_{s}$ for 0<ϑ<1 have been used to develop algorithms for recovering low-rank matrices in [19,20]. With this consideration, we let ${∥X∥}_{\vartheta \left(p,q\right)}^{\vartheta }=\sum_{i}\sigma_{i,\left(p,q\right)}^{\vartheta }$ and the substitution of the optimization (4) can be given as follows:

(5) $$\underset{X}{\arg\min}{∥X∥}_{\vartheta \left(p,q\right)}^{\vartheta }\;s.t.\;P_{\Omega }\left(X\right)=P_{\Omega }\left(A\right),$$

where 0<ϑ<1. We then propose an accelerated non-convex non-uniformed matrix completion algorithm (ANNMC) via variant quasi-norm optimization to adapt to the preference inference and eRRH/UE cluster partition for pre-caching in this section. The iteratively re-weighted framework and the accelerated framework are utilized for the algorithm design.

### 3.1. Nonconvex Nonuniformed Matrix Completion Agorithm

Inspired by the iterately re-weighted framework via $l_{1}$ norm in compressed sensing, an iterative procedure for solving the minimization problem (5) is as follows:

1. Set the iteration count t=1 and $w_{\tilde{i}}^{\left(0\right)}=1$, $\tilde{i}=1,\ldots ,N$.
2. Solve the weighted modified trace norm minimization problem
  (6) $$X^{\left(t\right)}=\underset{X}{\arg\min}\sum_{\tilde{i}}w_{\tilde{i}}^{\left(t-1\right)}\sigma_{\tilde{i},\left(p,q\right)}\;s.t.\;P_{\Omega }\left(X\right)=P_{\Omega }\left(A\right),$$
3. Update the weights: for each $\tilde{i}=1,\ldots ,N$,
  (7) $$w_{\tilde{i}}^{\left(t\right)}=\frac{1}{{\left(\sigma_{\tilde{i},\left(p,q\right)}^{\left(t\right)}+\varepsilon_{\tilde{i}}\right)}^{1-\vartheta }},$$
  where $\varepsilon_{\tilde{i}}>0$ in order to provide stability and to ensure that a zero-valued $\sigma_{\tilde{i}}^{\left(t\right)}\left(diag{\left(p\right)}^{1/2}Xdiag{\left(q\right)}^{1/2}\right)$ does not strictly prohibit a nozero estimate at the next step.
4. Terminate on convergence or when $t=t_{max}$. Otherwise, increment *t* and go to step 2.

In this method, using an iterative framework to construct the $w_{i}^{\left(t\right)}$ tends to allow for successively better estimation of the nonzero coefficients. Even though the early iterations may find inaccurate signal estimates, the largest signal coefficients are most likely to be identified as nonzero. Once these locations are identified, their influence is downweighted in order to allow more sensitivity for identifying the remaining small but nonzero signal coefficients.

### 3.2. Accelerated Agorithm for Convex Subproblem

Subsequently, we develop an accelerated variant for non-convex non-uniformed low-rank matrix completion algorithm. By referring optimization in [21], the subproblem (6) in iteration *t* can come down to

(8) $$\underset{X}{\arg\min}\tau \sum_{\tilde{i}}w_{\tilde{i}}^{\left(t-1\right)}\sigma_{\tilde{i},\left(p,q\right)}+\frac{1}{2}{∥X∥}_{F}^{2}\;s.t.\;P_{\Omega }\left(X\right)=P_{\Omega }\left(A\right).$$

Its Lagrangian function is defined as

(9) $$L\left(X,Y\right)=\tau \sum_{\tilde{i}}w_{\tilde{i}}^{\left(t-1\right)}\sigma_{\tilde{i},\left(p,q\right)}+\frac{1}{2}{∥X∥}_{F}^{2}+\langle Y,P_{\Omega }\left(X\right)-P_{\Omega }\left(A\right)\rangle ,$$

and its dual function is

(10) $$f\left(Y\right)=\underset{X}{\inf}L\left(X,Y\right).$$

We then intend to utilize the dual function (10) to solve the subproblem (8). To this end, We first deduce the properties of the dual function $f\left(Y\right)$ and then illustrate how to achieve the optimal solution of the subproblem (8) from its dual optimum directly. Now the following results should be given, which are essential to obtain the properties of $f\left(Y\right)$. We omit the proofs of the properties here and present them in Appendix A.

**Theorem** **2.** *For τ≥0, $Y\in R^{N\times K}$ and $w={\left\{w_{\tilde{i}}\right\}}_{\tilde{i}\in N^{+}}$, $0\leq w_{1}\leq \cdots \leq w_{N}$, the solution of the optimal problem $\min_{X}\frac{1}{2}{∥X-Y∥}_{F}^{2}+\tau \sum_{\tilde{i}}w_{\tilde{i}}\sigma_{i,\left(p,q\right)}$ obeys*

(11) $$D_{\tau ,w,\left(p,q\right)}\left(Y\right)=diag{\left(p\right)}^{1/2}U_{\left(p,q\right)}\Sigma_{\tau ,w,\left(p,q\right)}V_{\left(p,q\right)}^{T}diag{\left(q\right)}^{1/2},$$

*where $\Sigma_{\tau ,w,\left(p,q\right)}=diag{\left(\sigma_{\tilde{i}}\left(diag{\left(p\right)}^{-1/2}Ydiag{\left(q\right)}^{-1/2}\right)-\tau w_{\tilde{i}}\right)}_{+}$, and $diag{\left(p\;\right)}^{-1/2}Ydiag{\left(q\right)}^{-1/2}=U_{\left(p,q\right)}\Sigma_{Y,\left(p,q\right)}V_{\left(p,q\right)}^{T}$.*

Theorem 2 plays the crucial role in formulating the optimal of the subproblem (8). Additionally, $D_{\tau ,w,\left(p,q\right)}\left(•\right)$ is equal to $D_{\tau }\left(•\right)$ when vector $w=p=q=1={\left(1,\ldots ,1\right)}^{T}$, which is the crucial value for the traditional trace norm minimization solving. Based on the properties of Moreau-Yosida regularization and Theorem 2, we obtain the following result.

**Theorem** **3.** *For any $X,Y\in R^{N\times K}$, we have:*

(12) $${∥D_{\tau ,w,\left(p,q\right)}\left(X\right)-D_{\tau ,w,\left(p,q\right)}\left(Y\right)∥}_{F}^{2}\leq \langle D_{\tau ,w,\left(p,q\right)}\left(X\right)-D_{\tau ,w,\left(p,q\right)}\left(Y\right),X-Y\rangle ,$$

*which indicates that $D_{\tau ,w,\left(p,q\right)}\left(Y\right)$ is globally Lipschitz continuous with modulus 1.*

With Theorems 2 and 3, we obtain the following property of the dual function $f\left(Y\right)$.

**Theorem** **4.** *For any τ≥0, the dual function $f\left(Y\right)$ is continuously differentiable with Lipschitz continuous gradient at most 1, and the primal optimal $\hat{X}$ of the subproblem (8) is given by $\hat{X}=D_{\tau ,w,\left(p,q\right)}\left(P_{\Omega }\left(\hat{Y}\right)\right)$, when the dual optimal $\hat{Y}$ of the subproblem (8) is obtained.*

With the above properties, let $q\left(Y\right)=-f\left(Y\right)$. Since $f\left(Y\right)$ is the dual function of (8), $f\left(Y\right)$ is concave and subsequently $q\left(Y\right)$ is convex. Thus, for any $Y_{1},Y_{2}\in R^{N\times K}$, $\langle q\left(Y_{1}\right)-q\left(Y_{2}\right),Y_{1}-Y_{2}\rangle \geq 0$. From Equations (A8) and (A10) in Appendix A, it is also easy to show that $q\left(Y\right)$ satisfies $\nabla q\left(Y\right)=P_{\Omega }\left(D_{\tau ,w,\left(p,q\right)}\left(\left(Y\right)\right)-A\right)$ and belongs to $S_{0,1}^{1,1}\left(R^{N\times K}\right)$, which is the class of convex functions with Lipschitz gradient (i.e., for some 0≤μ≤1 and any $Y_{1},Y_{2}\in R^{N\times K}$, $q\left(Y\right)$ satisfies ${∥\nabla q\left(Y_{1}\right)-\nabla q\left(Y_{2}\right)∥}_{F}\leq {∥Y_{1}-Y_{1}∥}_{F}$ and $\langle \nabla f\left(Y_{1}\right)-\nabla f\left(Y_{2}\right),Y_{1}-Y_{2}\rangle \geq \mu {∥Y_{1}-Y_{2}∥}_{F}^{2}$). Therefore, optimization (8) can be solved by minimizing the objective function $q\left(Y\right)$, i.e.,

(13) $$\min_{Y}q\left(Y\right).$$

After acquiring this equivalent optimization $\min_{Y}q\left(Y\right)$, in the following we propose to solve this smooth convex optimization problem by using the Nesterov’s method, a very powerful optimization technique for class $S_{\mu ,L}^{1,1}\left(R^{N\times K}\right),\;\mu \geq 0,\;L<+\infty$ [22]. For $q\left(Y\right)$ belonging to $S_{0,1}^{1,1}\left(R^{N\times K}\right)$, The Nesterov’s method for this problem utilizes two sequences: $\left\{Y_{l}\right\}$ and $\left\{Z_{l}\right\}$, $Y_{l},Z_{l}\in R^{N\times K}$,

$$Z_{l}=Y_{l}+\beta_{l}\left(Y_{l}-Y_{l-1}\right),\;Y_{l+1}=Z_{l}-\frac{1}{L_{l}}\nabla q\left(Z_{l}\right).$$

where $\beta_{l}$ is a tuning parameter, and 11LlLl is the step size. By utilizing the Nemirovski’s line search scheme [23], which developed from the Nesterov’s method, for $L_{l}$ and $\beta_{l}$, the update scheme is that $L_{l+1}=2L_{l}$ and $\beta_{l}$ is independent on $L_{l}$. Starting from an initial point $Y_{0}$, $Z_{l}$ and $Y_{l+1}$ can be computed recursively, and arrive at the optimal solution $\hat{Y}$. We get Algorithm 1 to achieve the optimal solution of the subproblem (8) in the *t*-th iteration from its dual optimum directly. By using the Nesterov’s and Nemirovski’s scheme framework, the Algorithm 1 for the subproblem can achieve the convergence rate of O11tmax2tmax2.

**Algorithm 1** Accelerated Algorithm for Subproblem (6)**Input:**$\tilde{\mu },\alpha_{-1}=0.5,Y_{-1}^{\left(t\right)}=Y_{0}^{\left(t\right)}=Y_{n}^{\left(t-1\right)},L_{-1}=L_{0},\gamma_{0}\geq \tilde{\mu },\lambda_{0}=1,\vartheta ,\varepsilon_{\tilde{i}}>0$; **Output:**$Y_{n}^{\left(t\right)}$, $rank\left(Y_{n}^{\left(t\right)}\right)$;1:**for**l=0,1,2,…,n**do**2:   **while** true **do**3:      compute $\alpha_{l}\in \left(0,1\right)$ as the root of $L_{l}\alpha_{l}^{2}-\left(1-\alpha_{l}\right)\gamma_{l}-\alpha_{l}\tilde{\mu }=0$, $\gamma_{l+1}=\left(1-\alpha_{l}\right)\gamma_{l}+\alpha_{l}\tilde{\mu }$, $\beta_{l}=\frac{\left(1-\alpha_{l-1}\right)\gamma_{l}}{\left(\gamma_{l}+L_{l}\alpha_{l}\right)\alpha_{l-1}}$;4:      compute $Z_{l}^{\left(t\right)}=Y_{l}^{\left(t\right)}+\beta_{l}\left(Y_{l}^{\left(t\right)}-Y_{l-1}^{\left(t\right)}\right)$; $Y_{l+1}^{\left(t\right)}=Z_{l}^{\left(t\right)}-\frac{1}{L_{l}}\nabla q\left(Z_{l}^{\left(t\right)}\right)$; $L_{l}=2L_{l}$;5:   **end while**6:   $\lambda_{l+1}=\left(1-\alpha_{l}\right)\lambda_{l}$;7:**end for**8:**return**$Y_{n}^{\left(t\right)}$, $rank\left(Y_{n}^{\left(t\right)}\right)$;

We then summarize the proposed ANNMC algorithm and represent the execution steps. For the *t*-th iteration, we solve the subproblem (8) by Algorithm 1 to get a coarse result firstly. Since the entries of the objective preference matrix belong to the set $\left\{a_{1},a_{2},\ldots ,a_{I},0\right\}$, we add the quantification steps to ensure the result matrix in the feasible domain. To fit the observations better, the quantification thresholds are amended by the rate of value $a_{i}$ in the observations. Thus let the rate pai=NΩaiNΩaiNΩNΩ where $N_{\Omega }\left(a_{i}\right)$ is the number of $a_{i}$ in the observation and the quantification rule is shown in Algorithm 2. It aims to reduce the number of iterations as much as possible that the quantification is integrated in the *t*-th iteration but not after solving problem (5). In addition, the convergence of the ANNMC algorithm can be guaranteed due to the convergence of the iteratively re-weighted minimization for $l_{s}$ quasi-norm [19]. Specifically, because the ANNMC algorithm is based on the iteratively re-weighted framework and the $\left\{Y^{\left(t\right)}\right\}$ is generated by solving subproblem (8), there exists $\hat{Y}$, an accumulation point of $\left\{Y^{\left(t\right)}\right\}$, which is the first-order stationary point of problem (5). The method of the convergence proof is analogous to the method in [19,24], we omit it for conciseness.

There exists an additional remark that in practice the pre-mapping constructed by the proposed algorithms can only predict the preference-caching relations between UEs and eRRHs accurately with high probability due to the posteriori estimation. However, based on sufficient sampled data in a certain period, the preference/caching manner can be mining via the pre-mapping. The edge pre-caching strategy mentioned in Section 2 can facilitate the service with high QoS for users based on recommendation.

At the end of this section, we provide a concise example from an application to illustrate our algorithm at work. Assume that there exist *C* files prepared to be sent to eRRHs for pre-caching. Then, we execute the following steps to accomplish the task: (i) By recording the fragmentary request information of the UEs in the designated region, we first use the ANNMC algorithm to get the pre-mapping which concludes the UEs’ preference inferences and the corresponding eRRHs accessing information associated with the geographical influence. (ii) Since there are many intersection among the preference sets of different UEs in the same group, we merge these preference sets and regard the derived union as the criterion of the edge pre-caching. (iii) Classify the *C* files as *I* types according to the UEs’ preferences, and rank the file types and the files of each type respectively via the popularity in society and history of the UEs’ requests. (iv) Determine which eRRH cluster serves the corresponding UE group by using the pre-mapping. Then divide files into messages of the same size and deliver to the eRRHs based on the criterion of the edge pre-caching, until the cache capacity is exhausted. (V) If the pre-cached messages match the requests of the served UEs, the messages will be sent to the UEs and get the subsequent messages of the same files from BBU pool. Otherwise the fragmentary request information record of the UEs will be updated and then the pre-mapping is modified based on the ANNMC algorithm, and so on.

**Algorithm 2** ANNMC Algorithm**Input:**$\tilde{\mu },\alpha_{-1}=0.5,Y_{-1}=Y_{0},L_{-1}=L_{0},\gamma_{0}\geq \tilde{\mu },\lambda_{0}=1,\vartheta ,\varepsilon_{\tilde{i}}>0$; **Output:**$Y_{t_{max}}$, $rank\left(Y_{t_{max}}\right)$;  1:**for**$t=1,2,\ldots ,t_{max}$**do**  2:   solve subproblem (8) by Algorithm 1;  3:**end for**  4:**if** the (*k*,*j*)-th component of $Y_{t_{max}}$ satisfies      pai−1ai−1+paiaipai−1ai−1+paiaipai−1+paipai−1+pai≤Ytmaxj,k<paiai+pai+1ai+1paiai+pai+1ai+1pai+pai+1pai+pai+1,   wherepai=NΩaiNΩaiNΩNΩ and i=2,…,I;  5:   **then**  6:      ${\left(Y_{t_{max}}\right)}_{j,k}=a_{i}$;  7:**else**  8:      ${\left(Y_{t_{max}}\right)}_{j,k}=0$;  9:**end if**10:**return**$Y_{t_{max}}$, $rank\left(Y_{t_{max}}\right)$;

## 4. Energy Consumption Analysis

In light of mass data generated by billions of devices, we always prefer less energy consumption in data transmission, storing and processing. For this reason, edge caching aims to reduce repeated data transmission from original servers, which means that unnecessary energy consumption on packet delivery between the edge tier and the server tier can be saved. Moreover, caching itself also consumes extra energy while keeping RAM or disk memory running. To determine and sum up the overall cost of the entire simulation on the F-RAN network architecture, in summary, we may consider three parts of the energy consumption, the energy for device maintaining, content caching and transmission. Based on the content service strategy mentioned in Section 2 (also shown in Figure 2), we present the calculation of total energy consumption for each eRRH as follows:

(14) $$E_{total}=E_{device}+E_{cache}+E_{trans}.$$

Note that once the devices are deployed and work, the part to maintain all devices in the F-RAN can not be expressed in the total energy consumption since it is a fixed cost and can only be reduced by shutting down some devices [25]. Therefore, consider a fixed number of devices (eRRHs and users) in the F-RAN obeying the content per-caching strategy in Section 2 and all eRRHs are received and caches contents with the same size and number as the initial state. Then the total energy consumption analysis for each eRRH can be equivalent to the discussion on its total energy consumption change, which is the total energy cost for the content caching and the transmission for content update:

(15) $$E_{total\Delta }=E_{cache}+E_{trans\Delta }.$$

Especially, the caching energy cost

(16) $$E_{cache}=\eta E_{content},$$

where $E_{content}$ is the power consumption of keeping a content object in an eRRH and η is the is the size of the eRRH cache (the number of the content object). Meanwhile, since the content transmission energy cost of each eRRH contains the content sending and receiving energy cost by BBU pool and eRRH respectively, the transmission energy cost for the single content update $E_{content\Delta }=E_{send}+E_{recv}$. Then similar to the energy consumption linear model in [26,27],

(17) $$E_{trans\Delta }=\eta \left(\left(1-\theta \right)E_{content\Delta }+\delta \right),$$

where δ represents fixed costs and θ represents the rate of the required contents for the cluster users in the cache (i.e., 1−θ represents the content update rate).

With this energy consumption analysis model, we discuss $E_{total\Delta }$ under the condition upon the implement of the pre-mapping constructed by the proposed preference inference algorithm, i.e., ANNMC. For $E_{cache}$, one part of the $E_{total\Delta }$, because the inference algorithm with appropriate non-uniform sampling rate may accurately estimate the preference data of the cluster with high probability, the number of the caching contents η would be less than that of the blind caching. In other words, the accurate preference estimation may prompt the eRRH cluster cache only several certain kinds of contents but not as many kinds as possible to ensure the QoS of F-RAN. On the other hand, for $E_{trans\Delta }$, θ will be determined by the inference error of the preference data. Thus, the $E_{trans\Delta }$ directly associates with the accuracy of the proposed inference algorithm. Let $\eta_{min}$ denote the minimum number of cached content objects and $\eta_{max}$ denote the specific maximum number of contents allowed for caching, then the range of the total energy consumption change for each eRRH with the inference guidance is

(18) $$\eta_{\min}E_{content}\leq {\left.E_{total\Delta }\right|}_{infer}\leq \eta_{\max}\left(E_{content}+E_{content\Delta }+\delta \right).$$

Especially, with the low-rank assumption of the preference data and the proper low non-uniform sampling rate, the proposed ANNMC for inference can ensure the total energy consumption to approximate the lower bound with high probability, due to the foreseeable algorithm accuracy.

## 5. Performance Evaluations

The performance evaluations are illustrated in this section by using MATLAB. We first perform experiments on the synthetic networks, including static and dynamic cases, and show that our system model and the proposed pre-mapping construction method is feasible on the task of content preference distribution inference for the pre-caching in the F-RAN. Additionally, experimental simulations about the energy consumption performance of the pre-caching strategy are provided as well. To ensure that our results are reliable, we conduct all experiments 200 times, and average the results from all of the trials.

### 5.1. Performance of the Preference Inference

To verify the validity, we first consider a pre-mapping (complete preference matrix A) constructed by the synthetic F-RAN link data. The observation matrix $P_{\Omega }\left(A\right)$ is formed by sampling some entries from A. To be specific, for the F-RAN, we set several eRRH clusters. The clusters of UEs are formed on the basic pattern of the A and located randomly. Each UE cluster is served by one of eRRH cluster. Accordingly, A possesses complete diagonal-block structure which is mentioned and analysed in Section 2. The size of each UE cluster is larger than 20 and the sum of the sizes is K=1000 and the size of each eRRH cluster is larger than 5 and the sum of the sizes is N=200. Meanwhile, in the F-RAN the content types is sorted via popularity and its number I=15. For convenience, the preferred type subset of each UE cluster is sampled from the type set by Zipf distribution obeying the sorting. We further assume that only a part of requests are recorded by non-uniform sampling and the file types are valued by different integers (the max number of the content types contained by each eRRH cluster is set to be 5 as an example). The probability distribution of sampling ensures that each row (column) of the original matrix is sampled with different $p_{s}\in \left(0,1\right)$, and the practical sampling-rate of the original matrix A is defined as PΩA0,1A0,1A0,1A0,1, where ${∥•∥}_{0,1}$ is equal to the number of non-zero entries in the matrix. Then we use our proposed ANNMC algorithm to estimate the complete matrix A and compare the performance of our approach to traditional Alternating Least Square (tALS) [28], the weighted trace norm regularization (WTNR) [29] and Accelerated Singular Value Thresholding (ASVT) [30] for the content caching pre-mapping construction problem. The details of the ANNMC parameters are shown in Table 1, and the experimental settings of the compared methods are the same with the corresponding literatures.

**Figure 4:**
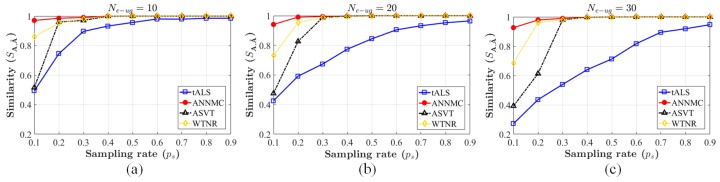
Comparison of similarity by different preference inferring algorithms (tALS, ASVT, WTNR and ANNMC) on the synthetic dataset. The number of blocks $N_{e-ug}=10$ corresponding to (**a**), 20 corresponding to (**b**) and 30 corresponding to (**c**). The range of the non-uniform sampling-rate $p_{s}$ is varied from 0.1 to 0.9 and the content types I=15.

**Figure 5:**
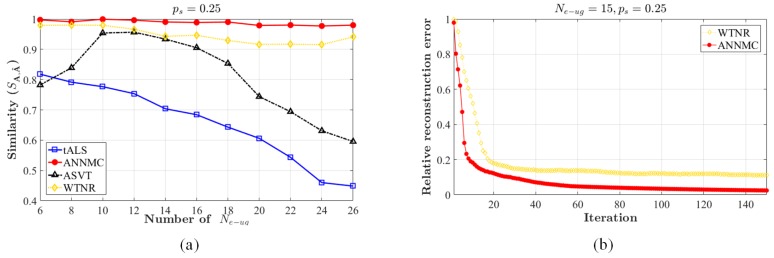
(**a**) shows the estimation similarity of the structure estimation for the preference matrix A with the number of the content types I=15, the number of groups $N_{e-ug}=6,8,\ldots ,26$ and the non-uniform sampling-rate $p_{s}=0.25$; (**b**) is Convergence rate of ANNMC and WTNR on the synthetic data A with the number of the content types I=15, $N_{e-ug}=15$ and the non-uniform sampling-rate $p_{s}=0.25$.

**Figure 6:**
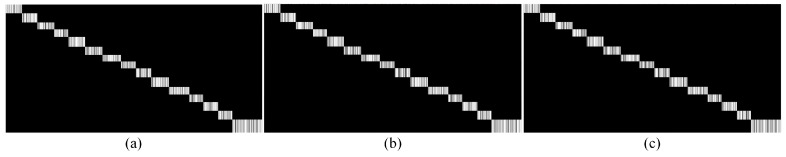
An visualized example of the pre-mapping construction for the preference matrix A ($N_{e-ug}\;=\;15$, I=15). (**a**) is the original preference matrix; (**b**) is the estimation result with 20% non-uniform samples (the similarity is 0.9942); (**c**) is the estimation result with 40% non-uniform samples (the similarity is 0.9991).

We evaluate the performances by the similarity between the inferred matrix $\hat{A}$ and the original matrix A to indicate the accuracy of estimation, the definition of which is SA,A^=A,A^A,A^AFA^FAFA^F. The range of the practical sampling-rate is from 0.1 to 0.9 and plot the inference accuracy in Figure 4. Apparently, the proposed non-uniform algorithm outperforms others due to higher accuracy. Moreover, we choose matrices (200×1000) with ranks r=6,8,…,24,26 (i.e., the number of the clusters) and non-uniform sampling rate $p_{s}=0.25$ for matrix completion. For each algorithm, we complete the structure with different $N_{e-ug}$ and compute recovery relative accuracy by similarity. The result is shown in Figure 5a. It is observed that ANNMC possesses the better robustness of non-uniform matrix completion in the certain range of eRRH/UE cluster number. We further compare ANNMC and WTNR due to their better estimation performance than the others in the simulation. As the results shown in Figure 5b, ANNMC outperforms WTNR with non-uniform sampling on low sampling-rate based estimation, even though its convergence rate is only slightly faster than WTNR. An visualized example of the pre-mapping constructed by ANNMC algorithm is also shown in Figure 6 ($N_{e-ug}=15$ and I=15 with sampling rates $p_{s}=0.2$ and $p_{s}=0.4$), which consists with our results.

### 5.2. Performance of the Dynamic Preference Inference

In general, the pre-mapping A varies during a time period long enough. Accordingly, the observations of this dynamic preference matrix over time essentially introduce time dimension to the problem of mining the potential eRRH/content-UE relationships. Therefore, a more realistic scenario is inferring the A by utilizing the historical records of the eRRH content caching and UEs’ requests in global and temporal evolvement perspectives. In particular, assume that we are given an incomplete non-uniformed observation tensor (or 3-dimensional array), which consists of the preference matrix pattern corresponding to the snapshots of the underlying dynamic relationships at time $T=T_{0}+1,T_{0}+2,\ldots ,T_{0}+\hat{T}$. Then the preference inference task is to estimate the possible pattern of the dynamic preference matrix at time $T_{0}+\hat{T}$ based on the given the 3-dimensional observation tensor. It is notice that, despite maintaining the dynamic property, the underlying eRRH/content-UE relationships in reality always display some “redundancy” attributed to the gradual periodic variation and the relatively stability [31]. With this property, the tensor consisting of the preference matrix patterns at *T* can be considered to be low-rank.

To construct synthetic networks for simulation, we first consider a complete dynamic eRRH/content-UE relationship network whose preference tensor is $A\in R^{I_{1}\times I_{2}\times I_{3}}$, $I_{1}\;=\;N,I_{2}\;=\;K,I_{3}=\hat{T}$. The slide of A at time *T* is an preference matrix pattern $A^{\left(T\right)}$ in the form of (1). In addition, for the gradual periodic variation, only a few kinds of $A^{\left(T\right)}$ (eRRH-UE group partition styles) exist in A and the patterns similar to each others are usually close in time. The observation tensor $P_{\Omega }\left(A\right)$ is formed by sampling some entries from A non-uniformly. Concretely, we let the F-RAN with the same settings in Section 5.1. Besides, for A, let the eRRH-UE cluster number be varied from 10 to 30 with step size 5, and for each cluster, 4 eRRH-UE group styles are generated randomly with Gaussian distribution as the basic styles. Then each basic style generates the other derived styles by perturbation and the total number of group style set is 40. We further assume that $I_{3}=\hat{T}=100$ and each group style appears randomly but more than once. The non-uniform sampling-rate of the original tensor A from 0.1 to 0.9 (it is realized by non-uniform sampling each slide with the given sampling-rate). Then with the given observed $P_{\Omega }\left(A\right)$, the task of the preference inference at time $T_{0}+\hat{T}$ is achieved by solving the following problem:

(19) $$\underset{X\in R^{I_{1}\times I_{2}\times I_{3}}}{\arg\min}\sum_{\hat{i}=1}^{3}{∥X_{\left(\hat{i}\right)}∥}_{\vartheta \left(p,q\right)}^{\vartheta }\;s.t.\;P_{\Omega }\left(X\right)=P_{\Omega }\left(A\right),$$

where $X_{\left(\hat{i}\right)}\in R^{I_{\hat{i}}\times I_{1}\cdots I_{\hat{i}-1}I_{\hat{i}+1}\cdots I_{3}}$ is the *k*th mode on X, and extracting the slide of X at the time $T_{0}+\hat{T}$ as the inferred pattern ${\hat{A}}^{\left(T_{0}+\hat{T}\right)}$.

**Figure 7:**
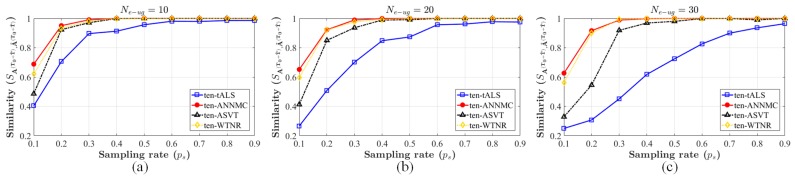
Comparison of similarity by different preference inferring algorithms for tensor completion (ten-tALS, ten-ASVT, ten-WTNR and ten-ANNMC) on the synthetic dataset. The number of blocks $N_{e-ug}^{\left(\hat{T}\right)}=10$ corresponding to (**a**), 20 corresponding to (**b**) and 30 corresponding to (**c**). The range of the non-uniform sampling-rate $p_{s}$ is varied from 0.1 to 0.9 and the content types I=15.

Similar with the approach in Section 3, we use ten-ANNMC, the variation of the algorithm (2) for tensor completion problem (19) to estimate ${\hat{A}}^{\left(T_{0}+\hat{T}\right)}$, and compare the performance of our approach to the variations of the same algorithms in Section 5.1 for tensor completion (ten-tALS, ten-ASVT and ten-WTNR). $S_{A^{\left(T_{0}+\hat{T}\right)},{\hat{A}}^{\left(T_{0}+\hat{T}\right)}}$, the similarity between the inferred matrix ${\hat{A}}^{\left(T_{0}+\hat{T}\right)}$ and the original setting $A^{\left(T_{0}+\hat{T}\right)}$, is utilized to indicate the accuracy of estimation as well. We implement the algorithms to infer one of the group patterns generated before with the eRRH-UE cluster number $N_{e-ug}^{\left(\hat{T}\right)}$ = 10, 20, 30 respectively and plot the inference accuracy in Figure 7. The inferred results is shown that our inference approach can almost be accurately estimated the objective group style and apparently outperforms the others. Meanwhile, the results for tensor completion case is worse than the matrix completion in Section 5.1 when the sampling-rate is low ($p_{s}=0.1$ and 0.2). This phenomenon is due to the fact that the inference based on tensor completion blends the the observation records of the different patterns with the ${\hat{A}}^{\left(T_{0}+\hat{T}\right)}$. However, this strategy is benefit for the content pre-caching since the inferred matrix pattern can partly integrate the features of the observation $A^{\left(T\right)}$ ($T=T_{0}+1,\ldots ,T_{0}+\hat{T}-1$) and be more likely to meet the requests.

### 5.3. Performance of the Energy Consumption

In this section, simulation results are provided to evaluate the energy consumption of the proposed cluster content caching strategy in the F-RAN. The terms for energy consumption simulation are consistent with the description in Section 4. Referring to the linear energy consumption model and the real energy measurements in [26], the power consumption of the single content caching for the eRRH and backhaul transmissions are set as $E_{content}=0.05$ W·sec and $E_{content\Delta }=0.24$ W·sec, respectively. Assume that the content type number *I* and $N_{cont}$, the number of the best popular contents corresponding to the different types, are given and large enough. Also, all of the eRRHs in one cluster cache the same contents to ensure the service quality. We then construct the complete preference matrix A according to the method mentioned in Section 5.1 at the beginning as reference. The contents cached in each eRRH cluster for UEs’ requests are determined by using pre-caching strategy in Section 2 (which can be presented by the value set of the corresponding block in A). Since the caching contents in this scenario are exactly matched the UEs’ requests without retransmission, the corresponding average total energy cost for each eRRH can be considered to be the minimum, i.e, $E_{min-total}=Aver\left\{0.05\eta_{\min}\right\}$ W·sec.

With the aforementioned assumptions, two pre-caching strategies are applied and compared for energy consumption analysis. The first one is the proposed edge caching/transmission strategy based on the content caching pre-mapping and description in Section 2. Due to the fact that η is the is the size of the eRRH cache (see Equation (16)) and $\eta \geq \eta_{\min}$, to improve the QoS for users, we will use the residual memory space to non-repetitively cache the contents by Zipf distribution, which obeys the popularity sorting for all contents. Subsequently, all cache in eRRH will be used and the average caching anergy cost is 0.05η W·sec. Furthermore, note that the probability of retransmission is closely associated with the accuracy of the A inferred by ANNMC, we set θ is equal to the similarity between the $\hat{A}$ and the A, i.e., $S_{A,\hat{A}}$. Therefore, with (16) and (17), the average total energy cost for each eRRH based on the proposed strategy is presented as $Aver\left\{{\left.E_{total\Delta }\right|}_{infer}\right\}=\left(0.05+0.24\left(1-\theta \right)\right)\eta$ W·sec (δ in (17) is omitted since it is extremely smaller than the other terms). To compare performances, the traditional uniform per-caching strategy is given for simulation. For the traditional one, the active eRRHs in the F-RAN utilize all caches to retain the same content set $S_{\eta }$ selected by Zipf distribution obeying the popularity sorting for all contents. Hence, while the $E_{cache}$ is equal to that of the proposed strategy, the retransmission energy cost is $Aver\left\{0.24\eta_{retrans}\right\}$ W·sec where $\eta_{retrans}=\left|S_{m}-S_{\eta }\right|$. Then $Aver\left\{{\left.E_{total\Delta }\right|}_{trad}\right\}=\left(0.05\eta +0.24Aver\left\{\left|S_{m}-S_{\eta }\right|\right\}\right)$ W·sec. Both of the strategies are compared with the minimum consumption $E_{min-total}$, which represents the lower bound.

**Figure 8:**
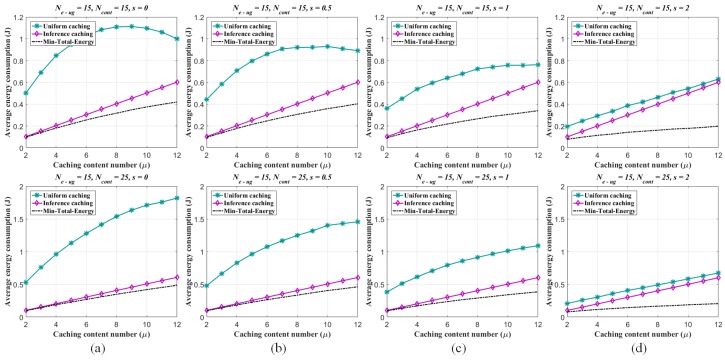
Average energy consumption of each eRRH in the F-RAN on the synthetic dataset (measured as *J* due to J= W·sec). Two different edge caching strategies are considered: (i) uniform caching strategy via content Zipf distribution, (ii) inferring caching strategy via pre-mapping (inferred by ANNMC with non-uniform sampling rate $p_{s}=0.2$) and Zipf distribution. The average energy consumptions of both strategies are compared with the minimum consumption $E_{min-total}$. The range of the caching content number of each eRRH is varied from 2 to 12. The Zipf exponent s=0,0.5,1,2, and (**a**–**d**) correspond to different *s* with $N_{e-ug}=15$, $N_{cont}=15$ and 25.

**Figure 9:**
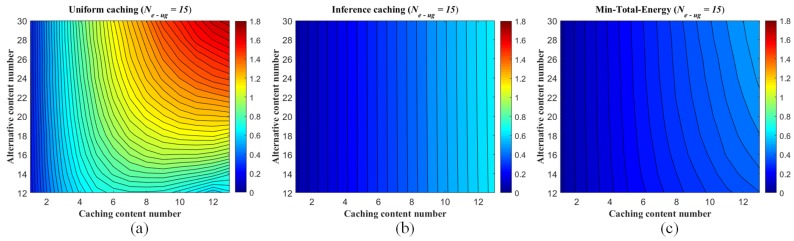
Average energy consumption range of each eRRH in the F-RAN on the synthetic dataset (measured as *J*) with the setting that $N_{e-ug}=15$ and Zipf exponent s=0.5. (**a**) is the uniform caching strategy via content Zipf distribution; (**b**) is the proposed inferring caching strategy via pre-mapping (inferring by ANNMC with non-uniform sampling rate $p_{s}=0.2$) and Zipf distribution; (**c**) is the minimum consumption $E_{min-total}$.

The energy consumption performances of the per-caching strategies are evaluated as follows. For one thing, given the numbers of the UEs and eRRHs respectively (K=1000, N=200) and fixed the UE/eRRH cluster number ($N_{e-ug}=15$), the number of the all alternative contents $N_{cont}$ and the Zipf exponent *s* are changed respectively ($N_{cont}=15,25$; s=0,0.5,1,2). The non-uniform sampling rate for pre-mapping construction by ANNMC is set to be $p_{s}=0.2$ and the corresponding inference similarity $S_{A,\hat{A}}=0.9942$. The average energy consumptions generated by the different number of caching contents in each eRRH are illustrated in Figure 8. By given $N_{e-ug}=15$ and s=0.5, the trends of the energy consumption via the changes of the caching content number and the alternative content number are shown in Figure 9 as well. As shown in the figures, the proposed caching strategy via the pre-mapping inference emerges better performance of the energy efficiency than the traditional one.

**Figure 10:**
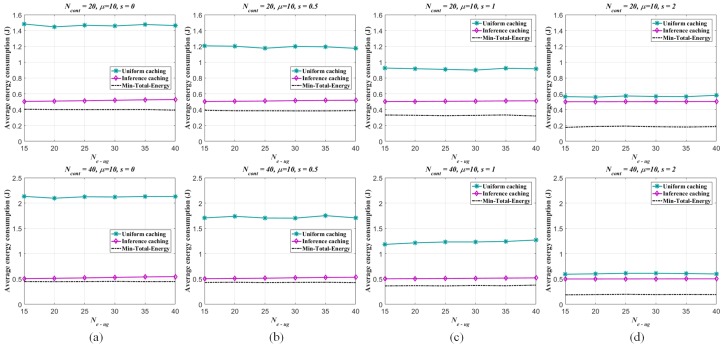
Average energy consumption of each eRRH in the F-RAN on the synthetic dataset (J= W·sec). Two different edge caching strategies are considered: (i) uniform caching strategy via content Zipf distribution, (ii) inferring caching strategy via pre-mapping (inferred by ANNMC with non-uniform sampling rate $p_{s}=0.2$) and Zipf distribution. The average energy consumptions of both strategies are compared with the minimum consumption $E_{min-total}$. The range of the eRRH/UE cluster number is varied from 15 to 40. The Zipf exponent s=0,0.5,1,2, and (**a**–**d**) correspond to different *s* with μ=10, $N_{cont}=20$ and 40.

**Figure 11:**
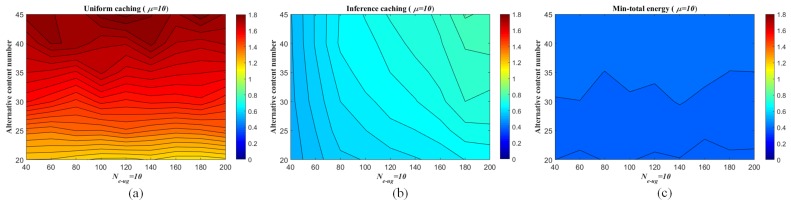
Average energy consumption range of each eRRH in the F-RAN on the synthetic dataset (measured as *J*) with the setting that μ=10 and Zipf exponent s=0.5. (**a**) is the uniform caching strategy via content Zipf distribution; (**b**) is the proposed inferring caching strategy via pre-mapping (inferring by ANNMC with non-uniform sampling rate $p_{s}=0.2$) and Zipf distribution; (**c**) is the minimum consumption $E_{min-total}$.

Meanwhile, the energy efficiency performance of the traditional strategy is approximating to the caching strategy via inference as *s* increasing, since the user interests converge to fewer popular content objects stored in the BBU pool. For the other, we fix $N_{cont}$ and the number of caching contents in each eRRH (μ) and show the energy consumption associated with the number of the UE/eRRH clusters in Figure 10. While the better performance of the proposed strategy and the similar properties related to the Zipf exponent *s* are illustrated in this figure, the average energy consumption of the proposed strategy slightly increases as the UE/eRRH cluster number is enlarged (i.e., the size of the cluster is decreased), since the rank of the matrix (i.e., $N_{e-ug}$) effects the inferring accuracy by using ANNMC ($S_{A,\hat{A}}=0.9954,0.9908,0.9866,0.9799,0.9749,0.9712$ when $N_{e-ug}$ changes from 15 to 40 with 5 interval). Also, due to the user interests converging to fewer popular content objects when *s* is increasing as well, the average energy consumption increasing trend is weaken. Furthermore, by given μ=10, s=0.5 and varied $N_{e-ug}$ form 40 to 200, the trends of the energy consumption via the changes of the caching content number and the UE/eRRH cluster number are shown in Figure 11. It illustrates that the energy efficiency performance is weaken along with the increasing of the caching content number and the UE/eRRH cluster number. Especially, fixing the caching content number, the worst energy efficiency performance of our proposed strategy is almost reached when the UE/eRRH cluster number meets the maximum (i.e., $N_{e-ug}=200$).

## 6. Conclusions

In this paper, based on digging out the non-uniform observed users’ network behavior information, we propose an edge content pre-caching strategy in F-RANs to improve the QoS for the users and energy efficiency. Especially, we analyze the relationships among the users’ activity, the content requesting and the state of the eRRH caching, and established pre-mapping for users’ content preference inferring by an accelerated non-convex matrix completion algorithm with the non-uniform observations. The dynamic scenario is also discussed and the developed variation based on tensor completion possesses good performance for the inferring task. In addition, the energy consumption analysis is given to reveal the properties of the pre-mapping based caching strategy. The simulation results show that the inferring algorithm for pre-mapping construction (preference inferring) is effective. Meanwhile, the inferring-based caching strategy possesses the advantages in energy saving compared to traditional uniform caching via Zipf distribution.

## Figures and Tables

**Table 1:** The parameter settings of the ANNMC.

Parameter	τ	ϑ	$\varepsilon_{i}$	$\tilde{\mu }$	$\alpha_{-1}$	$L_{0}$	$\lambda_{0}$
Setting	$2\sqrt{NK}$	0.5	$10^{-7}$	0.1	0.5	psps1.11.1	1

## Appendix A

**Proof of Theorem 2.** Let $\Sigma =diag\left(\sigma_{\tilde{i},X}\right)$ and $\Sigma_{\left(p,q\right)}=diag\left(\sigma_{\tilde{i},\left(p,q\right)}\right)$, which implies the nonzero entries of the diagonal matrices are in non-increasing order. Since the penalty term only depends on the singular values of $diag{\left(p\right)}^{1/2}Xdiag{\left(q\right)}^{1/2}$, problem $\min_{X}\frac{1}{2}{∥X-Y∥}_{F}^{2}+\tau \sum_{\tilde{i}}w_{\tilde{i}}\sigma_{\tilde{i},\left(p,q\right)}$ can be equivalently written as

(A1) $$\min_{\Sigma_{\left(p,q\right)}}\left\{\min_{X}\left(\frac{1}{2}{∥X-Y∥}_{F}^{2}+\tau \sum_{\tilde{i}}w_{\tilde{i}}\sigma_{\tilde{i},\left(p,q\right)}\right)\right\}.$$

For the inner minimization, due to von Neumann’s trace inequality, we have

$$\begin{array}{cc} \frac{1}{2}{∥Y-X∥}_{F}^{2} & =tr\left(YY^{T}\right)-2tr\left(YX^{T}\right)+tr\left(XX^{T}\right)\\ & =tr\left(\Sigma_{Y}^{2}\right)-2tr\left(diag{\left(p\right)}^{-1/2}Ydiag{\left(q\right)}^{-1/2}{\left(diag{\left(p\right)}^{-1/2}Xdiag{\left(q\right)}^{-1/2}\right)}^{T}\right)+tr\left(\Sigma^{2}\right)\\ & \geq tr\left(\Sigma_{Y}^{2}\right)-2tr\left(\Sigma_{Y,\left(p,q\right)}\Sigma_{\left(p,q\right)}\right)+tr\left(\Sigma^{2}\right). \end{array}$$

Especially the equality holds when $diag{\left(p\right)}^{1/2}Xdiag{\left(q\right)}^{1/2}$ admits the singular value decomposition $diag{\left(p\right)}^{1/2}Xdiag{\left(q\right)}^{1/2}=U_{\left(p,q\right)}\Sigma_{\left(p,q\right)}V_{\left(p,q\right)}^{T}$, where $U_{\left(p,q\right)}$ and $V_{\left(p,q\right)}$ are defined as the left and right singular matrices of $p^{-1/2}Yq^{-1/2}$. Meanwhile $X=U\Sigma V^{T}$, where U and V are defined as the left and right singular matrices of Y. Then the optimization reduces to

(A2) $$\min_{\Sigma_{\left(p,q\right)}}\left\{\frac{1}{2}tr\left(\Sigma^{2}\right)-tr\left(\Sigma_{Y,\left(p,q\right)}\Sigma_{\left(p,q\right)}\right)+\tau tr\left(diag\left(w_{\tilde{i}}\right)\Sigma_{\left(p,q\right)}\right)+\frac{1}{2}tr\left(\Sigma_{Y}^{2}\right)\right\}.$$

Note that $diag{\left(p\right)}^{1/2}Xdiag{\left(q\right)}^{1/2}=diag{\left(p\right)}^{1/2}U\Sigma V^{T}diag{\left(q\right)}^{1/2}$, i.e.,

(A3) $$\Sigma =U^{T}diag{\left(p\right)}^{1/2}U_{\left(p,q\right)}\Sigma_{\left(p,q\right)}V_{\left(p,q\right)}^{T}diag{\left(q\right)}^{1/2}V,$$

the objective function is completely separable and is minimized only when

(A4) $$\nabla_{\Sigma_{\left(p,q\right)}}\left(\frac{1}{2}tr\left(\Sigma \Sigma^{T}\right)-tr\left(\Sigma_{Y,\left(p,q\right)}\Sigma_{\left(p,q\right)}\right)+tr\left(\tau diag\left(w_{\tilde{i}}\right)\Sigma_{\left(p,q\right)}\right)\right)=0,$$

where $\nabla_{\Sigma_{\left(p,q\right)}}\left(•\right)$ denotes the gradient for $\Sigma_{\left(p,q\right)}$. Utilizing $\nabla_{X}tr\left(A^{T}\mathrm{XB}\right)=AB^{T}$ and $\nabla_{X}tr\left(\mathrm{AXB}X^{T}\right)=A^{T}XB^{T}+\mathrm{AXB}$, we have

$$\begin{array}{c} U_{\left(p,q\right)}^{T}diag{\left(p\right)}^{1/2}U\left(U^{T}diag{\left(p\right)}^{1/2}U_{\left(p,q\right)}\Sigma_{\left(p,q\right)}V_{\left(p,q\right)}^{T}diag{\left(q\right)}^{1/2}V\right)V^{T}diag{\left(q\right)}^{1/2}V_{\left(p,q\right)}\\ ={\left(\Sigma_{Y,\left(p,q\right)}-diag\left(\tau w_{\tilde{i}}\right)\right)}_{+}, \end{array}$$

i.e.,

(A5) $${\;U}_{\left(p,q\right)}^{T}diag{\left(p\right)}^{1/2}Xdiag{\left(q\right)}^{1/2}V_{\left(p,q\right)}={\left(\Sigma_{Y,\left(p,q\right)}-diag\left(\tau w_{\tilde{i}}\right)\right)}_{+}$$

Therefore $D_{\tau ,w,\left(p,q\right)}\left(Y\right)=p^{1/2}U_{\left(p,q\right)}{\left(\Sigma_{Y,\left(p,q\right)}-diag\left(\tau w_{\tilde{i}}\right)\right)}_{+}V_{\left(p,q\right)}^{T}q^{1/2}$ is a global optimal solution to the optimization problem. The uniqueness of the solution follows by the equality condition for the von Neumann’s trace inequality when has distinct nonzero singular values, and the uniqueness of the strictly convex optimization (A2). This concludes the proof. □

**Proof of Theorem 4.** Since $L\left(X,Y\right)=\tau \sum_{\tilde{i}}w_{\tilde{i}}^{\left(t\right)}\sigma_{\tilde{i},\left(p,q\right)}+\frac{1}{2}{∥X∥}_{F}^{2}+\langle Y,P_{\Omega }\left(X\right)-P_{\Omega }\left(A\right)\rangle$,

(A6) $$\begin{array}{cc} f\left(Y\right) & =\underset{X}{\inf}L\left(X,Y\right)=\underset{X}{\inf}\left(\tau \sum_{\tilde{i}}w_{\tilde{i}}^{\left(t\right)}\sigma_{\tilde{i},\left(p,q\right)}+\frac{1}{2}{∥X∥}_{F}^{2}+\langle Y,P_{\Omega }\left(X\right)-P_{\Omega }\left(A\right)\rangle \right)\\ & =\underset{X}{\inf}\left(\tau \sum_{\tilde{i}}w_{\tilde{i}}^{\left(t\right)}\sigma_{\tilde{i},\left(p,q\right)}+\frac{1}{2}{∥X∥}_{F}^{2}+\langle Y,P_{\Omega }\left(X\right)\rangle -\langle Y,P_{\Omega }\left(A\right)\rangle \right)\\ & =\underset{X}{\inf}\left(\tau \sum_{\tilde{i}}w_{\tilde{i}}^{\left(t\right)}\sigma_{\tilde{i},\left(p,q\right)}+\frac{1}{2}{∥X-P_{\Omega }\left(Y\right)∥}_{F}^{2}\right)+\langle Y,P_{\Omega }\left(A\right)\rangle -\frac{1}{2}{∥P_{\Omega }\left(Y\right)∥}_{F}^{2}\\ & =g\left(Y\right)+\langle Y,P_{\Omega }\left(A\right)\rangle -\frac{1}{2}{∥P_{\Omega }\left(Y\right)∥}_{F}^{2} \end{array}$$

For $g\left(Y\right)$, using the well-known properties of Moreau-Yosida Regularization [32], we get the results that $g\left(Y\right)$ is a globally continuously differentiable convex function. Moreover, $\nabla g\left(Y\right)=P_{\Omega }\left(Y-D_{\tau ,w,\left(p,q\right)}\left(P_{\Omega }\left(Y\right)\right)\right)$ and $\nabla g\left(Y\right)$ is continuously differentiable with Lipschitz continuous gradient 1, i.e., for any $Y_{1},Y_{2}\in R^{N\times K}$,

(A7) $${∥\nabla g\left(Y_{1}\right)-\nabla g\left(Y_{2}\right)∥}_{F}\leq {∥P_{\Omega }\left(Y_{1}\right)-P_{\Omega }\left(Y_{2}\right)∥}_{F}\leq {∥Y_{1}-Y_{2}∥}_{F}.$$

Then the gradient of $f\left(Y\right)$ can be obtained as follows:

(A8) $$\begin{array}{cc} \nabla f\left(Y\right) & =\nabla g\left(Y\right)+P_{\Omega }\left(A\right)-P_{\Omega }\left(Y\right)\\ & =P_{\Omega }\left(Y-D_{\tau ,w,\left(p,q\right)}\left(P_{\Omega }\left(Y\right)\right)\right)+P_{\Omega }\left(A\right)-P_{\Omega }\left(Y\right)\\ & =P_{\Omega }\left(A-D_{\tau ,w,\left(p,q\right)}\left(P_{\Omega }\left(Y\right)\right)\right). \end{array}$$

It follows that for any $Y_{1},Y_{2}\in R^{N\times K}$,

(A9) $$\begin{array}{cc} {∥\nabla f\left(Y_{1}\right)-\nabla f\left(Y_{2}\right)∥}_{F} & ={∥P_{\Omega }\left(A-D_{\tau ,w,\left(p,q\right)}\left(P_{\Omega }\left(Y_{1}\right)\right)\right)-P_{\Omega }\left(A-D_{\tau ,w,\left(p,q\right)}\left(P_{\Omega }\left(Y_{2}\right)\right)\right)∥}_{F}\\ & ={∥P_{\Omega }\left(D_{\tau ,w,\left(p,q\right)}\left(P_{\Omega }\left(Y_{1}\right)\right)-D_{\tau ,w,\left(p,q\right)}\left(P_{\Omega }\left(Y_{2}\right)\right)\right)∥}_{F}\\ & \leq {∥D_{\tau ,w,\left(p,q\right)}\left(P_{\Omega }\left(Y_{1}\right)\right)-D_{\tau ,w,\left(p,q\right)}\left(P_{\Omega }\left(Y_{2}\right)\right)∥}_{F}. \end{array}$$

Following from Theorem 3 and Cauchy-Schwarz inequality, we have

(A10) $${∥\nabla f\left(Y_{1}\right)-\nabla f\left(Y_{2}\right)∥}_{F}\leq {∥P_{\Omega }\left(Y_{1}\right)-P_{\Omega }\left(Y_{2}\right)∥}_{F}\leq {∥Y_{1}-Y_{2}∥}_{F}.$$

When the dual optimal $\hat{Y}$ is obtained, by using the result of Equation (A6), we can get

(A11) $$\begin{array}{cc} \hat{X} & =\arg\min_{X}L\left(X,Y\right)=\arg\min_{X}\left(\tau \sum_{\tilde{i}}w_{\tilde{i}}^{\left(t\right)}\sigma_{\tilde{i},\left(p,q\right)}+\frac{1}{2}{∥X-P_{\Omega }\left(\hat{Y}\right)∥}_{F}^{2}\right)\\ & =D_{\tau ,w,\left(p,q\right)}\left(P_{\Omega }\left(\hat{Y}\right)\right). \end{array}$$

This concludes the proof. □

## References

[1] Zikria Y., Kim S., Afzal M., Wang H., Rehmani M. (2018). 5G Mobile services and scenarios: Challenges and solutions. Sustainability.

[2] Checko A., Christiansen H.L., Yan Y., Scolari L., Kardaras G., Berger M.S., Dittmann L. (2015). Cloud RAN for mobile networks—A technology overview. IEEE Commun. Surv. Tutor..

[3] Peng M., Wang C., Lau V., Poor H.V. (2015). Fronthaul-constrained cloud radio access networks: Insights and challenges. IEEE Wirel. Commun..

[4] Wang K., Li X., Ji H., Du X. (2016). Modeling and optimizing the LTE discontinuous reception mechanism under self-similar traffic. IEEE Trans. Veh. Technol..

[5] Wang K., Yu X., Lin W., Deng Z., Liu X. (2019). Computing aware scheduling in mobile edge computing system. Wirel. Netw..

[6] Park S.H., Simeone O., Sahin O., Shitz S.S. (2014). Fronthaul compression for cloud radio access networks: Signal processing advances inspired by network information theory. IEEE Signal Process. Mag..

[7] Peng M., Yan S., Zhang K., Wang C. (2015). Fog-computing-based radio access networks: Issues and challenges. IEEE Netw..

[8] Bi S., Zhang R., Ding Z., Cui S. (2015). Wireless communications in the era of big data. Commun. Mag. IEEE.

[9] Park S.H., Simeone O., Shitz S.S. (2016). Joint optimization of cloud and edge processing for fog radio access networks. IEEE Trans. Wirel. Commun..

[10] Peng X., Shen J.C., Zhang J., Letaief K.B. Backhaul-aware caching placement for wireless networks. Proceedings of the 2015 IEEE Global Communications Conference.

[11] Tandon R., Simeone O. Cloud-aided wireless networks with edge caching: Fundamental latency trade-offs in fog radio access networks. Proceedings of the 2016 IEEE International Symposium on Information Theory (ISIT).

[12] Tao M., Chen E., Zhou H., Yu W. (2016). Content-centric sparse multicast beamforming for cache-enabled cloud RAN. IEEE Trans. Wirel. Commun..

[13] Sengupta A., Tandon R., Simeone O. (2017). Fog-aided wireless networks for content delivery: Fundamental latency tradeoffs. IEEE Trans. Inf. Theory.

[14] Zhao Z., Peng M., Ding Z., Wang W., Poor H.V. (2016). Cluster content caching: An energy-efficient approach to improve quality-of-service in cloud radio access networks. IEEE J. Sel. Areas Commun..

[15] Shanmugam K., Golrezaei N., Dimakis A.G., Molisch A.F., Caire G. (2013). FemtoCaching: Wireless content delivery through distributed caching helpers. IEEE Trans. Inf. Theory.

[16] Candes E.J., Recht B. (2009). Exact matrix completion via convex optimization. Found. Comput. Math..

[17] Keshavan R.H., Montanari A., Oh S. (2009). Matrix completion from noisy entries. J. Mach. Learn. Res..

[18] Boumal N., Absil P.A. (2015). Low-rank matrix completion via preconditioned optimization on the Grassmann manifold. Linear Algebra Appl..

[19] Lai M.J., Xu Y., Yin W. (2013). Improved iteratively re-weighted least squares for unconstrained smoothed ∖ell_q minimization. SIAM J. Numer. Anal..

[20] Marjanovic G., Solo V. (2012). On *l*_*q* optimization and matrix completion. IEEE Trans. Signal Process..

[21] Cai J.F., Candès E.J., Shen Z. (2010). A singular value thresholding algorithm for matrix completion. SIAM J. Optim..

[22] Nesterov Y. (2013). Introductory Lectures on Convex Optimization: A Basic Course.

[23] Nemirovski A. (2005). Efficient Methods in Convex Programming.

[24] Cen Y., Cen Y., Wang K., Li J., Chen S., Zhang L., Tao D. Low-rank tensor estimation via generalized norm/quasi-norm difference regularization. Proceedings of the 2018 4th International Conference on Big Data Computing and Communications (BIGCOM).

[25] Gabry F., Bioglio V., Land I. (2016). On energy-efficient edge caching in heterogeneous networks. IEEE J. Sel. Areas Commun..

[26] Feeney L.M., Nilsson M. Investigating the energy consumption of a wireless network interface in an ad hoc networking environment. Proceedings of the Twentieth Annual Joint Conference of the IEEE Computer and Communications Societies (INFOCOM 2001).

[27] Xu J., Ota K., Dong M. (2018). Saving energy on the edge: In-memory caching for multi-tier heterogeneous networks. IEEE Commun. Mag..

[28] Hsieh C.J., Chiang K.Y., Dhillon I.S. Low rank modeling of signed networks. Proceedings of the 18th ACM SIGKDD International Conference on Knowledge Discovery and Data Mining.

[29] Salakhutdinov R., Srebro N. Collaborative filtering in a non-uniform world: Learning with the weighted trace norm. Proceedings of the 23rd International Conference on Neural Information Processing Systems-Volume 2.

[30] Hu Y., Zhang D., Liu J., Ye J., He X. Accelerated singular value thresholding for matrix completion. Proceedings of the 18th ACM SIGKDD International Conference on Knowledge Discovery and Data Mining.

[31] Dunlavy D.M., Kolda T.G., Acar E. (2011). Temporal link prediction using matrix and tensor factorizations. ACM Trans. Knowl. Discov. Data (TKDD).

[32] Hiriart-Urruty J.B., Lemaréchal C. (2013). Convex Analysis and Minimization Algorithms I: Fundamentals.

